# A simple tool to automate the insertion process in cochlear implant surgery

**DOI:** 10.1007/s11548-020-02243-7

**Published:** 2020-08-28

**Authors:** Thomas S. Rau, M. Geraldine Zuniga, Rolf Salcher, Thomas Lenarz

**Affiliations:** 1 Department of Otolaryngology, Hannover Medical School, Carl-Neuberg-Str. 1, 30625 Hannover, Germany; 2 Cluster of Excellence EXC 2177/1 “Hearing4all”, Hannover Medical School, Hannover, Germany

**Keywords:** Cochlear implant, Automated insertion, Insertion tool, Hydraulic actuation

## Abstract

**Purpose:**

Automated insertion of electrode arrays (EA) in cochlear implant surgery is presumed to be less traumatic than manual insertions, but no tool is widely available in the operating room. We sought (1) to design and create a simple tool able to automate the EA insertion process; and (2) to perform preliminary evaluations of the designed prototype.

**Methods:**

A first prototype of a tool with maximum simplicity was designed and fabricated to take advantage of hydraulic actuation. The prototype facilitates automated forward motion using a syringe connected to an infusion pump. Initial prototype evaluation included: (1) testing of forward motion at different velocities (2) EA insertion trials into an artificial cochlear model with force recordings, and (3) evaluation of device handling, fixation and positioning using cadaver head specimens and a surgical retractor. Alignment of the tool was explored with CT imaging.

**Results:**

In this initial phase, the prototype demonstrated easy assembly and ability to respond to hydraulic actuation driven by an infusion pump at different velocities. EA insertions at an ultra-slow velocity of 0.03 mm/s revealed smooth force profiles with mean maximum force of 0.060 N ± 0.007 N. Device positioning with an appropriate insertion axis into the cochlea was deemed feasible and easy to achieve.

**Conclusions:**

Initial testing of our hydraulic insertion tool did not reveal any serious complications that contradict the initially defined design specifications. Further meticulous testing is needed to determine the safety of the device, its reliability and clinical applicability.

## Introduction

A cochlear implant (CI) is a neuroprosthesis containing an electrode array (EA), which is implanted or introduced into the human inner ear (cochlea) to electrically stimulate the auditory nerve for auditory rehabilitation of patients with severe to profound sensorineural hearing loss. The most crucial step of the whole surgery—the implantation of the EA into the cochlea—is characterized by the mechanical interaction of the “foreign body” with the surrounding intracochlear tissues. Inserting the “foreign body” into the intracochlear biological environment may cause injuries to the delicate soft tissue structures, with the basilar membrane being the most critical as it holds the sensory cells of hearing [1]. Therefore, traumatic insertions can result in loss of residual hearing [2]. Moreover, preservation of residual hearing is highly beneficial as many investigations have described improved hearing outcomes [3, 4] when combining electric stimulation (ES) using the cochlear implant with the natural, residual acoustic (A) hearing. To date, this strategy is widely known as electric acoustic stimulation (EAS) [5–7].

Motivated by these findings, prevention of intracochlear trauma became a dominant topic in cochlear implantation surgery since the 1990s. In order to reduce intracochlear trauma and improve hearing outcomes, Lehnhardt introduced the concept of the “soft surgery technique” already in 1993 [8]. However, reliable prevention of intracochlear trauma remains a clinical challenge, as the damage resulting from the EA insertion process is influenced by multiple, interdependent factors that are difficult to control. Furthermore, their direct effect on trauma and functional outcome remains difficult to be fully understood, as these factors do not occur isolated from each other. These include the surgical approach (e.g., angle of insertion trajectory, size and location of the opening into the inner ear, level of surgical skills), the type of EA (e.g., stiffness properties, cross-sectional area, surface smoothness), as well as the insertion technique (e.g., insertion velocity). Automation of the insertion process could minimize intracochlear trauma as it allows for:

- standardization, independent of EA characteristics or surgical expertise
- smoothing and optimization, eliminating human tremor and jitter [9, 10]
- deceleration, enabling ultra-slow continuous insertion velocities beyond those which are humanly feasible [11, 12].

Initial works on automation of EA insertions emerged in the late 1990s and early 2000s in laboratory settings and consisted on motor-driven insertions of EA prototypes that sought to standardize electrode insertion trials and enhance comparability and reproducibility of results [13–15]. This feed-forward automation was later integrated to robotic insertions of custom-made steerable electrodes [16, 17]. In those works, during the insertion process the EA is accurately controlled by actuation to facilitate active adaption of the EA’s shape to the patient-specific, spiral-shaped lumen of the inner ear.

Later, the concept of an automated insertion tool to use with standard electrodes in the context of CI surgery was published by Hussong et al. [18]. However, a clinical application was not conducted, leaving out a realistic description of its sterile use. Our further research was focused on implementation of force sensing capabilities [19, 20] leading to an increase in complexity due to the integration of more sensitive electronical components. Therefore, development of a solution for sterile use of these tools was disregarded.

More recently, other groups have also developed motorized insertion tools in laboratory experiments [21–23] and although they were also not designed for use under sterile conditions, valuable findings have resulted from these works [9, 11, 21, 24–29]. For example, automated EA insertions have shown its capability in reducing critical peak insertion forces [9, 10, 30]—subsequently promising to reduce insertion trauma. Different insertion velocities have been explored using automated setups, and to date, most data suggest that lower insertion velocities produce lower insertion forces [11]. Moreover, these automated insertions can be programmed to occur at speeds slower than 0.9 mm/s, which according to the work of Kesler et al. [12] are not manually feasible as a continuous, steady movement.

Recently, two approaches for robotic surgical systems developed for clinical-, intraoperative use have been described. The robotics-assisted surgical tool proposed by Kaufmann et al. has already published promising results in its pre-clinical validation phase [10], but has the drawback of requiring additional drilling and screwing to the patient’s head to fixate the tool. In addition, a teleoperated robot (RobOtol, Collin Orl, Bagneux, France) has been used by Nguyen et al. to perform EA insertions in first patients [31]. However, this system requires the use of a robotic arm, which may significantly increase CI surgery costs and potentially limit patient access to this technology.

As an alternative, we introduce a new concept of a simple tool designed to automate the feed-forward motion of EAs or intracochlear catheters in CI surgery. Its design aims to achieve a maximum simplicity to facilitate wide clinical and surgical translation while still complying with surgical necessities (e.g., sterility).

The scope of this manuscript is to (1) present the ideation and design details of the tool, herein after referred to as Cochlea Hydro Drive (CHD), and (2) test the defined main features such as automation capabilities, handling and adaptation to a surgical-like scenario.

## Materials and methods

The design process included the following requirements specification:

- To facilitate an automated feed-forward motion serviceable for implantation of EAs or intra-cochlear catheters [32] into the inner ear.
- To operate at different slow velocities, particularly ultra-slow, down to at least 0.03 mm/s [11].
- Easy adaptation to EAs or cochlear catheters from different manufacturers.
- To meet sterility needs and regulations (as it is intended for intraoperative use).
- To be stabilized and provide alignment along a patient-specific insertion trajectory without additional invasive procedures.
- To remain simple to allow its wide future use.

### CHD prototype design

The design of the CHD repurposes a commercially available, sterile, disposable syringe as hydraulic cylinder. This provides automated hydraulic actuation. The plunger of the syringe (Omnifix 5 ml Luer Lock Solo, B. Braun Melsungen AG, Germany) serves as a piston and transforms the pressure inside the barrel into a continuous, steady, linear and feed-forward movement. The pressure to run the CHD is delivered by an infusion pump (Injectomat 2000, Fresenius Kabi, Bad Homburg, Germany) located outside the sterile boundary and connected via a flexible, standard sterile tube. For experimental evaluation, water was used as fluid, but saline or sterile water may also be used. This shifting of the main components for generation and control of the motorized movement to outside the sterile area avoids the need for sterilization or sterile draping of electromechanical components and is a key element of the achieved simplification in comparison with previously described tools [19–21, 33]. Insertion velocity can be controlled indirectly via the infusion pump by setting a corresponding flow rate. In the current setting (5 ml syringe, infusion pump with flow rate between 0.1 and 400 ml/h), insertion velocity can be in the range of 0.0002–0.94 mm/s. A 3-way stopcock valve (Discofix, B. Braun) was added to the tubing as an additional safety feature. It allows the surgeon or surgical assistant to immediately stop the movement of the CHD. Furthermore, the valve is used to vent the tubing.

Sterile adaptors were designed and built for the connection of the syringe with an external positioning device and its connection to the device considered for implantation. A U-shaped holder, made of stainless steel, was designed as an interchangeable piece to be adapted or replaced to hold different types of implantable devices. In its current version, the holder was adapted to the extracochlear outer diameter of MED-EL’s EA series and MED-EL’s cochlear catheter. Silicone replicas of such devices were used for some initial trials and are henceforth simply called “probes”. The probe holder is mounted using a split adaptor to the plunger flange. As the probe is held using a form-fit connection, the probe will need to be offloaded manually of the U-shaped holder (e.g., using tweezers).

A syringe holder clamps the barrel and connects it via a stainless steel rod with the positioning device. The split adapter and the syringe holder are made of polyether ether ketone (PEEK), a polymer known to be thermostable and used to produce high-quality plastic parts for medical devices. All custom-made parts were fabricated using conventional manufacturing processes (milling, cutting) and can be sterilized (e.g., steam sterilization) before being delivered to the operating room, where the surgical team can then assemble the tool.

**Fig. 1:**
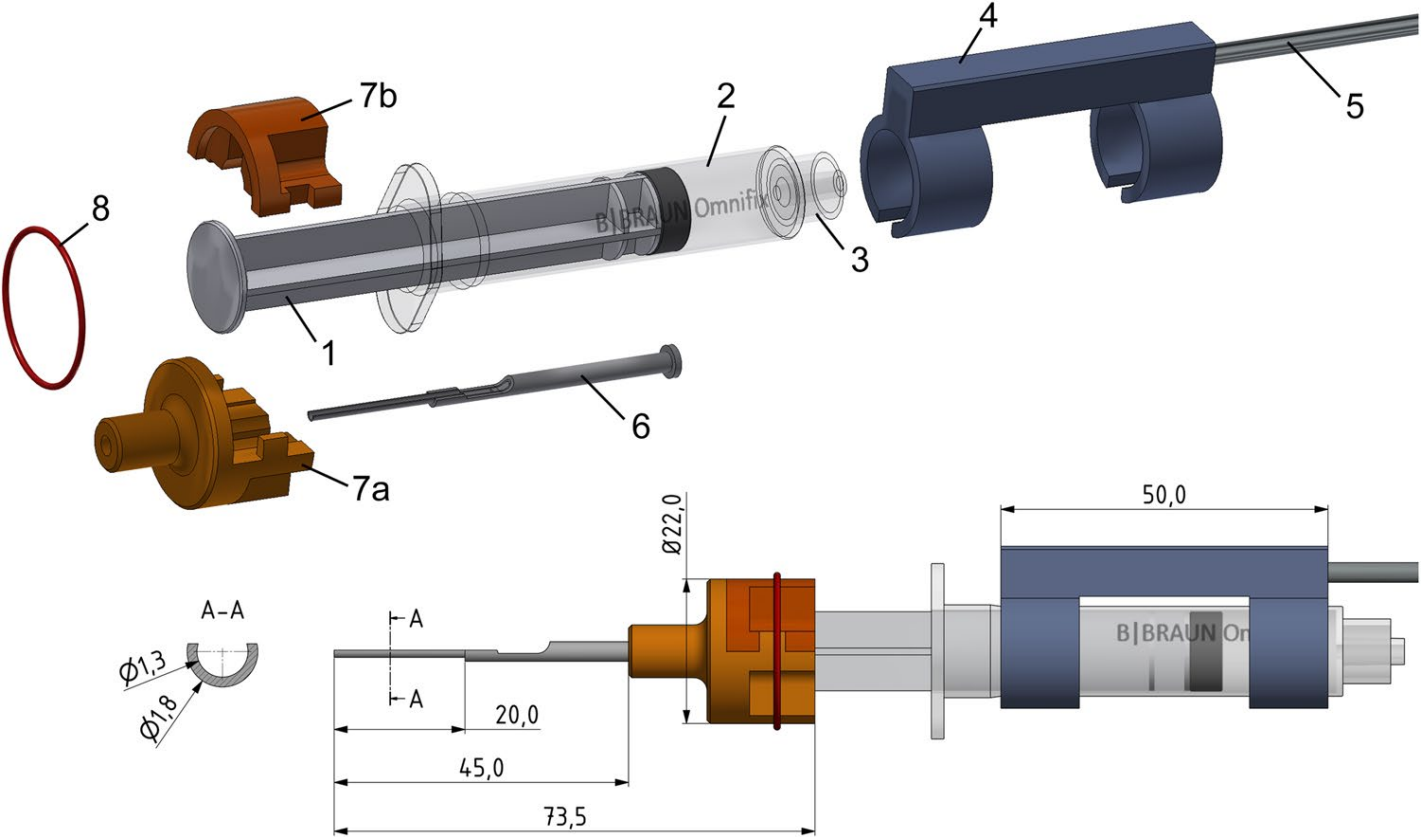
Schematic drawing of the CHD. The tool consists of the following parts: 1 plunger, 2 barrel and 3 luer lock connector of the syringe, 4 syringe holder, 5 stainless steel rod, 6 U-shaped probe holder, 7a and 7b split adapter to connect the probe holder with the plunger. The two separate parts of the adapter are equipped with a lip feature and secured with a silicone ring (8). All dimensions are in millimeters

The prototype is designed to be connected to a standard surgical retractor with a flexible arm (Flexible Arm Cerebellar Retractor, #50-1520, Codman, Raynham, MA, US) for positioning (Fig. 1). The flexible arm needs to be adjusted manually by the surgeon under direct visual control, and surgical experience and judgment is then needed to achieve an optimal position.

### Initial testing

#### Evaluating the automated feed-forward mechanism

**Fig. 2:**
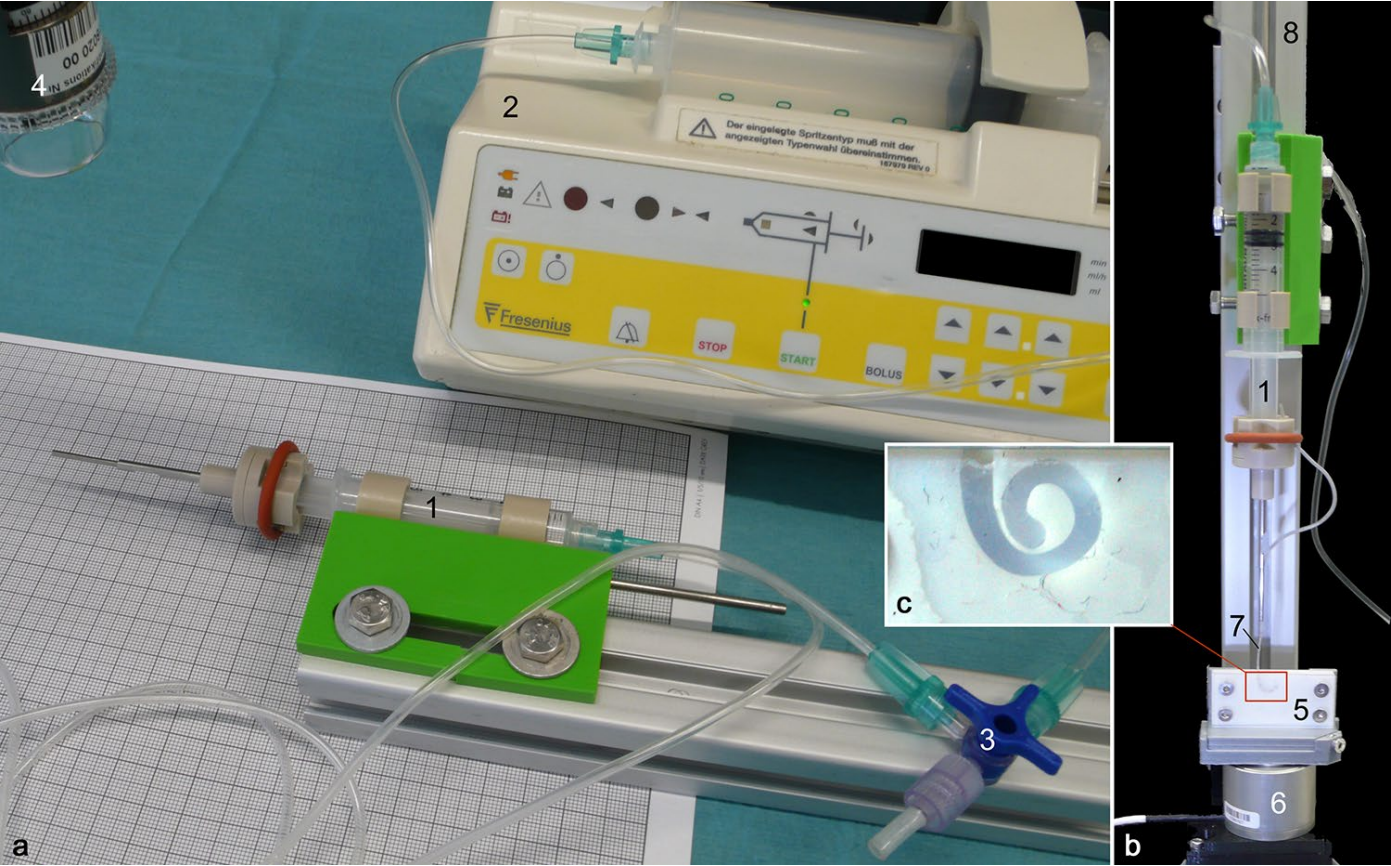
**a** Test bench for testing the hydraulic actuation of the probe holder at different velocities. CHD (1) connected to the infusion pump (2). A 3-way Stopcock valve (3) serves to vent the tubing and as an emergency stop. Microscope camera (4) to capture the movement of the probe holder. **b** Test bench for insertion force measurement. The aCM (5) is fixed on top of a load cell (6). The CHD enables hydraulic actuation of the EA (7) into the model while being fixed to a stand (8). **c** Inset showing the aCM geometry

To test the concept of a hydraulic feed-forward mechanism, the CHD was assembled and fixed on a test bench (Fig. 2a). The distal motion of the U-shaped holder in front of the plunger was captured by a microscopic camera. The infusion pump was connected using the described tubing and valve, they were flushed with water as described above, and finally the pump was programmed to run at 170 ml/h, 46.8 ml/h and 12.8 ml/h which correspond to 0.4 mm/s, 0.11 mm/s and 0.03 mm/s, respectively. For each velocity, ten trials were conducted, while the movement of the probe holder in front of millimeter paper was video recorded. All videos were qualitatively assessed regarding a continuous, feed-forward movement without breaks and quantitatively analyzed regarding the average resulting velocity from moving a distance of 10 mm, 3 mm and 2 mm, respectively. Attention was paid to determine a significant delay between the stop of the infusion (using the stop valve) and actual cessation of movement.

#### Insertion force measurements

To test its main functional goal, 10 insertions of a straight EA (STANDARD, MED-EL, Innsbruck, Australia) into an artificial cochlea model (aCM) were performed using our tool. A force measurement setup was used to characterize the force profile and smoothness of the forward movement of the CHD during the insertions (Fig. 2b).

The CHD was fixed above the aCM, which was mounted on top of a load cell (K3D35, ME-Meßsysteme GmbH, Hennigsdorf, Germany). The aCM was one of our standard models representing the geometry of the first full turn of an average sized human scala tympani. The cochlear lumen (with lateral wall length of 27 mm) was milled out of a polytetrafluoroethylene (PTFE, a.k.a. “Teflon”) sheet and filled with saline solution for realistic friction conditions [34]. The infusion pump was set to infusion rates corresponding to 0.03 mm/s and 0.40 mm/s, which are insertion velocities practically impossible to the human hand in a continuous fashion [12]. At the beginning of each recording, the EA was placed at the opening of the aCM and was inserted approximately 24.5 mm until it reached the end of the aCM. After each trial, the EA was carefully straightened by hand. A custom-made software was used to read out the force sensor every 25 ms with a measuring amplifier (GSV-4USB-D37, ME-Meßsysteme GmbH) including analog–digital converter (16 bit). No filter was applied to the force data. More details about our artificial cochlea models and the force measurement setup have been described elsewhere [11, 35].

#### Evaluating the tool in a surgical-like scenario

To evaluate if the CHD can indeed be fixated with a noninvasive approach, two formalin-fixed, previously anonymized, human cadaver heads were used under approval of the authors’ institutional review board. Different strategies to position the CHD using a surgical retractor with a flexible arm were tried for both ears, including different options to fix the retractor and different orientations of the flexible arm. The goal was to fixate the CHD allowing it to aim toward the facial recess and round window area based on judgement by a CI surgeon with the aid of a microscope. Positioning of the CHD in front of the round window was repeated 10 times for each right and left ears.

Additionally, these trials were used to obtain an initial impression of the desired workflow as well as to test assembly time of the CHD, including mounting of the probes. Cone-beam computed tomography imaging was performed for one of the trials after positioning of the CHD to objectively illustrate the alignment of the tool along an appropriate insertion axis.

## Results

**Fig. 3:**
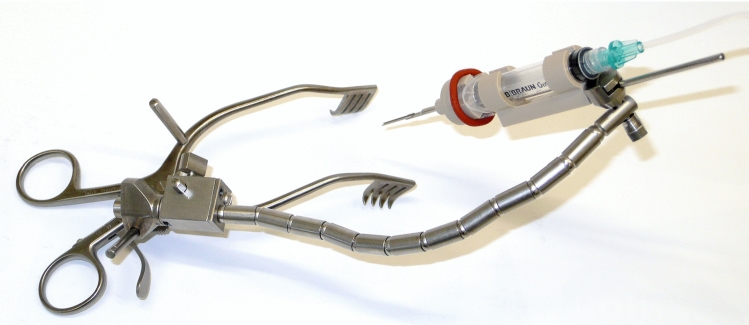
Current prototype after assembly

Based on the general idea of utilizing a standard, sterile, single-use syringe as a hydraulic cylinder to provide an automated, forward linear movement for implantation of intra-cochlear probes, a first prototype was designed (Fig. 1) and manufactured (Fig. 3).

### Preliminary results of testing the hydraulic actuation

A total of 30 trials were completed and revealed a slow and smooth forward movement. There was a varying delay between turning the valve and start of the hydraulic actuation, most likely due to some remaining, compressible air bubbles in the tubing. No observable jerks or stops once the movement started were noted. The measured average velocity was (0.420 ± 0.038) mm/s, (0.072 ± 0.007) mm/s and (0.050 ± 0.007) mm/s for the tested flow rates (170 ml/h, 46.8 ml/and 12.8 ml/h, respectively), differing slightly to the expected feed-forward velocities of 0.4 mm/s, 0.11 mm/s and 0.03 mm/s, respectively. Furthermore, the cessation of movement of the holder occurred immediately after the stop of the infusion using the valve.

### Insertion behavior and force profiles

**Fig. 4:**
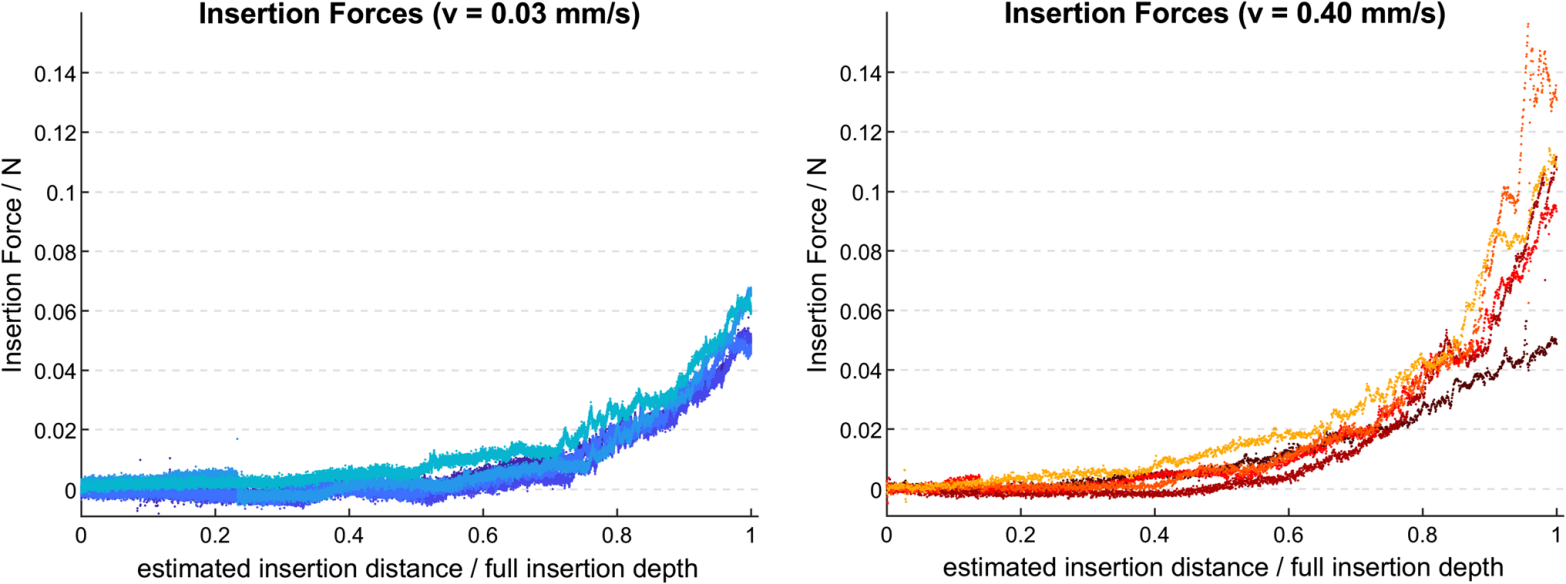
Insertion forces for ultra-slow (left) and low (right) insertion velocities using the CHD. Estimated insertion distance presented in percentage of full insertion depth to normalize across all observations

Figure 4 depicts insertion force profiles using the CHD. Mean maximum forces were (0.060 ± 0.007) N when the infusion rate targeted an insertion velocity of 0.03 mm/s, and (0.107 ± 0.036) N for targeted 0.4 mm/s. Force profiles in Fig. 4 are normalized using insertion depths estimated in percentage to normalize across all trials. The smooth increase in force as the EA is inserted deeper is comparable to those observed with other automated setups [11, 36].

### Findings from cadaver trials

**Fig. 5:**
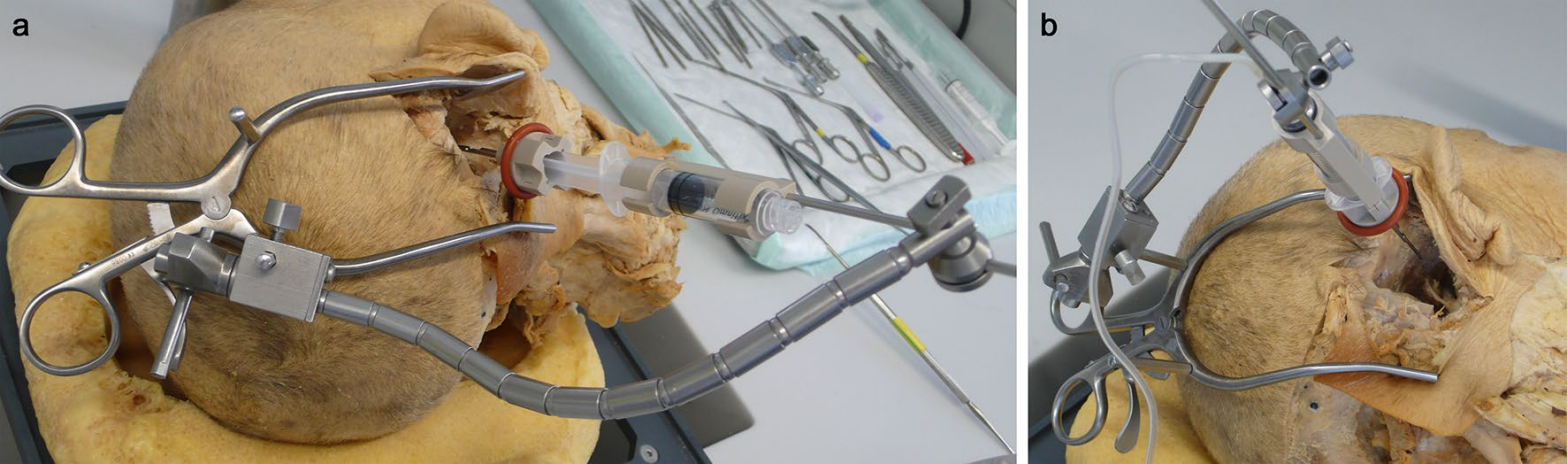
Mounting of the CHD to the cadaver’s head. Different positions were tested aiming the tip of the instrument deep toward the cochlea. The flexible arm provides a sufficient stable configuration

**Fig. 6:**
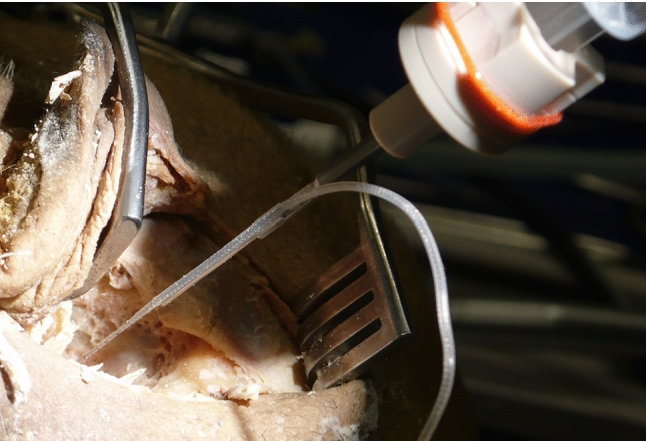
CHD loaded with EA

Assembly of the prototype and its application on human cadaveric head specimens was easy and reproducible. Assembly time for the CHD is less than 1 min (approximately 30 s), while fixation of the retractor, mounting of the flexible arm and the CHD as well as positioning of the device takes 10–15 min. An otolaryngology, head and neck surgeon (MGZ) who was not involved in the initial device design was able to handle and position the device accordingly. Positioning of the device along an adequate trajectory to enter the inner ear was possible for all trials (Fig. 5 and 6) and confirmed under surgical visualization using the microscope.

**Fig. 7:**
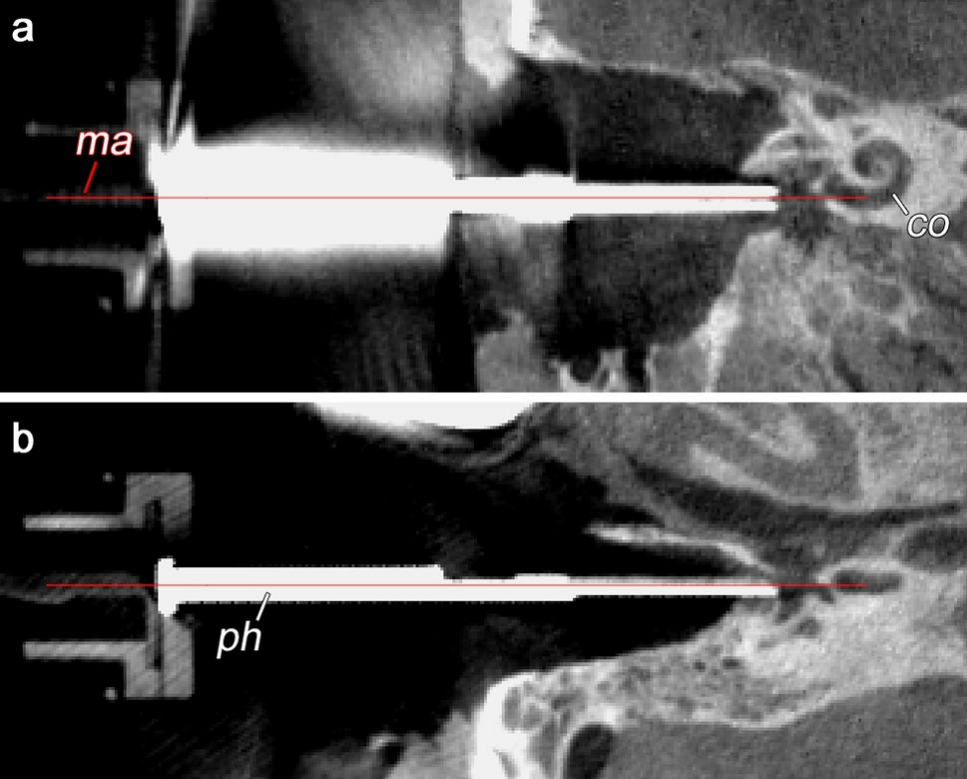
CBCT image of the CHD in its final position aiming toward the cochlea (co). Main axis (ma) of the probe holder (ph) is visualized (red line)

The cone-beam computed tomography (CBCT) imaging performed for one of the trials illustrates the proper alignment axis of our tool with the round window membrane and basal turn of the cochlea (Fig. 7). The tool was able to stand alone using the flexible arm of the retractor (Fig. 5) without visible drift due to gravity or other loads for a period of at least 20 min. Loading of the probes (after good positioning was achieved) into the U-shaped holder was also doable, and these did not fall off the holder in these trials. Good visualization of the instrument, tip of the instrument and tip of the probe was achieved using a binocular microscope. The test runs did not reveal major complications that contradict further development of the tool toward its clinical application.

## Discussion

The here presented prototype fulfills conformity with all needs for sterility. This is achieved by a strong simplification of the concept and the reduction to the essential: use of a standard infusion pump, a disposable commercially available syringe and a few small size adapters. By repurposing a syringe as a hydraulic cylinder, one can avoid sterilization or sterile housing of an electrically powered actuator, such as stick–slip piezo drives [18], or rotary motor [21] as previously reported. All components for energy generation and motion control are located outside the sterile area of the OR as they are integrated in the infusion pump. Furthermore, standard infusion pumps are already optimized for providing very low but also very precise flow rate, which can be adjusted in a wide range.

Our initial testing shows that repurposing a syringe as a hydraulic cylinder facilitates hydraulic actuation. A feed-forward motion without significant peaks or breaks was observed for all three programmed velocities. We observed deviations in the resulting average velocity compared to the theoretical calculated values. This is better appreciated in the trials corresponding to 0.11 mm/s, where the resulting velocity was 0.07 mm/s. However, this finding does not contradict the general concept as these results still fall within the desired range of “ultra-slow” velocities. Additionally, accuracy in setting the velocity of the plunger can be improved by further testing and calibration of the system and re-evaluation of the workflow.

Insertions of an EA into an aCM confirmed that our tool is indeed able to hold and insert an EA in response to hydraulic actuation. These trials showed smooth force profiles, especially with an ultra-slow velocity of 0.03 mm/s, which in turn resulted in mean maximum forces of 0.060 ± 0.007 mN (Fig. 4a). The mean insertion forces resulting from our experiments are comparable to previous reports using MED-EL electrodes [36, 37]. However, caution is warranted when comparing our tool’s absolute force values with other studies, as different methodologies pertaining to the cochlea model (geometry, material, lubricant), EA and insertion depth may also impact the resulting insertion forces. Therefore, the most valuable finding from the present insertion experiments using the CHD is that the resulting insertion profiles do not show numerous peaks, pauses or breaks, in contrast to manually performed insertions, as previously reported in [9, 12, 30].

In case of leakage in the tubing or connectors only a part of the fluid flow will reach the CHD which causes a decrease in the insertion velocity (or even a full stop of the movement depending on the size of the leak). This may be an argument to use sterile or saline water instead of regular water in the operating room, therefore avoiding an additional risk for the patient.

Incorporation of the 3-way stopcock valve allows for sufficient venting of the tubing. When that few small air bubbles remained inside the valve, no impact on the movement of the device was observed. Furthermore, this allows for an immediate cessation of feed-forward motion of the CHD when used to stop the insertion.

In its current design, automated backward motion after implantation is not possible. The plunger needs to be moved backwards (inside the syringe of the CHD) manually or indirectly by removing the larger syringe (of the syringe driver) and pulling its plunger in order to generate a negative pressure. The 5 ml syringe allows for a travel range of approx. 45 mm which covers full insertion depth of even the longest EA in the market (with 31.5 mm). Until now, our tool was developed for straight (lateral-wall) EAs.

A drawback of the simplicity of the CHD is that a force sensing capability is not integrated as in previous tools [19, 20]. Therefore, monitoring of the insertion process based on insertion forces is not possible in the current design of the CHD. However, we consider this as an acceptable limitation, since the current version of the tool is designed to facilitate automation of the EA insertion and potentially allow for very slow insertion speeds and therefore reduced insertion forces [11]. In addition, the current design of the CHD allows for its use with the conventional mastoidectomy with a facial recess approach, which is an “open” surgery access to the inner ear. Thereby, the surgeon can still visually observe the insertion process and stop the automated feed of the implant if irregularities (e.g., EA buckling) occur. One has to investigate in further studies whether the loss of the surgeon’s capability to manually sense at least some insertion forces [38] is a critical issue; especially as the soft surgery protocol suggests cessation of the insertion when resistance is met. Additional investigations are required to elucidate whether visual observation of the insertion process ensures the same safety for the residual hearing as the haptic feedback. This is of particular importance in patients with functional residual hearing.

The implementation of a tool like the CHD could bring other benefits. For example, the device could later on be combined with additional intraoperative measurements such as fluoroscopy [39], or cochlear monitoring [40] in order to achieve a deeper understanding of the electrode insertion process and the underlying mechanism of intracochlear trauma. Ultimately, more information and strategies could be gathered to develop different approaches that could guarantee preservation of residual hearing.

In its current version, the tool is intended to be positioned using a flexible arm attached to a standard surgical retractor. This requires sufficient experience of the surgeon in estimating the best trajectory into the basal turn of the cochlea for the individual patient [41]. However, this is also true in conventional, manually performed electrode insertion and therefore not a specific challenge when using the CHD. In addition, using the surgical retractor, automation of probe insertions goes without additional invasive steps, such as screwing into the skull, as required in the case of the iotaMotion system in order to drive the unit [10].

In case of using a tool like the CHD for EA insertion, the alignment to an individually planned trajectory can be improved by incorporating image-guided surgery systems [22, 42, 43] or micro-stereotactic frames [44, 45] into the surgical setting. In doing so, an optimized trajectory can be planned based on patient-specific imaging and followed in the OR with an accuracy outperforming what is manually feasible. This is another possibility that can be further explored in the future.

The limitations of this work include that we did not perform probe insertions into human cadaveric cochlear specimens with the use of the CHD. However, we have explored our initial concept to justify moving forward with such experiments. Also, the learning curve of assembling, handling and positioning of the tool is herein not fully characterized, but will be further explored. Likewise, the variability on the resulting velocities at which the CHD response needs to further validated and replicated.

To summarize, more detailed investigations have to address questions such as: How accurately can the device be positioned manually along an appropriate insertion axis? Is there a steep learning curve or how does these results depend on the experience of the surgeons? What about the inter-operator variability when mounting, positioning and using the CHD? How reliably can the actual insertion of the EAs into the cochlea be performed using the tool? How do different velocities impact insertion trauma? However, answering these questions is reserved for further studies.

## Conclusion

Initial testing of our hydraulic insertion tool did not reveal any serious complications that contradict the initially defined design specifications. Further meticulous testing is needed to determine the safety of the device, its reliability and clinical applicability.
